# Effects of adding neuromuscular electrical stimulation to functional training on muscle recruitment, pain reduction, and knee joint function in patellofemoral pain syndrome patients

**DOI:** 10.1097/MD.0000000000036095

**Published:** 2024-01-19

**Authors:** Qiu Nie, Yaming Yu, Zheng Yuan, Jian Chen

**Affiliations:** a Affiliated Hospital of North Sichuan Medical College, Nanchong, Sichuan, China; b Sichuan Orthopaedic Hospital, Chengdu, Sichuan China; c Sichuan Orthopaedic Hospital, Chengdu, Sichuan China; d Wuhan Sports Institute, Wuhan, Hubei, China.

**Keywords:** muscle recruitment, neuromuscular electrical stimulation, patellofemoral pain syndrome, VMO

## Abstract

**Background:**

Impaired lower extremity motor function and knee pain are common concerns in patients with patellofemoral pain syndrome (PFPS). It is essential to plan therapeutic techniques to therapy PFPS. The objective of this study was to determine the effect of neuromuscular electrical stimulation (NMES) combined with functional training on pain, lower extremity function and muscle recruitment in patients with PFPS.

**Methods:**

Twenty-four PFPS patients (male-13, female-11) were selected to conduct this study finally. Two groups were formed: the control group (n = 12) which included functional training only and the experimental group (n = 12) which functional training along with NMES-in both groups interventions were performed for 45 minutes/session, 3 days/weeks for 6 weeks. The functional training consisted of warm-up activities, strength training, balance training, and relaxation activities. All patients were evaluated with surface electromyography testing system for the root mean square and integrated electromyography of vastus medial oblique (VMO), vastus lateralis (VL), and VMO/VL ratio, visual analog scores (VAS) for pain, and Kujala functional score for knee joint function before and after 6 weeks. Normality was tested for all outcome variables using Shapiro–Wilk test. Nonparametric (Mann–Whitney *U* test and Wilcoxon signed-rank test) tests were used to analyze data. A 2-way analysis of variance with repeated measures (group*time) was applied to analyze the data.

**Results:**

A significant increases (*P* < .001) root mean square and integrated electromyography of VMO, VMO/VL ratio, and Kujala score in both groups, For VAS scores, significant decreases was observed in both groups. When both groups were compared, greater improvement (*P* < .05) was observed in the experimental group in comparison the control group for both knee pain, lower extremity function and muscle recruitment. However, there was no difference in VL muscle recruitment between the 2 groups.

**Conclusions:**

Functional training and NMES combined with functional training are helpful to improve pain, knee function and muscle recruitment of PFPS patients. NMES combined with functional training was more effective compared to the control group. This may help clinical trialists to use different NMES to synchronize other interventions in future studies to enhance rehabilitation efficacy in PFPS patients through passive training versus active stimulation.

## 1. Introduction

Patellofemoral pain syndrome (PFPS) is one of the most common knee joint injuries, characterized by anterior or peripatellar pain resulting from pathological or biomechanical abnormalities of the patellofemoral joint. It is commonly provoked by functional activities such as running, jumping, squatting, stair climbing, and prolonged sitting with flexed knees.^[1]^ Epidemiological studies have shown that PFPS primarily affects young individuals aged 18 to 35 years, particularly adolescent athletes, with an annual incidence rate of approximately 22 per 1000 individuals.^[2]^ Without timely and effective treatment, Long-term follow-up studies have revealed that 45% of patients initially diagnosed with PFPS subsequently develop osteoarthritis, significantly impairing their daily activities and quality of life.^[3]^ Possible mechanisms for the development of PFPS have been found to be related to joint pain, which in turn leads to reflexive inhibition of the periarticular muscles, atrophy of lower extremity muscles (especially vastus medial oblique [VMO] and gluteus maximus [GMAX]), and reduction of muscle strength, muscle imbalance, creating a vicious cycle that further exacerbates pain and continues to reduce knee function and cause long-term discomfort. In addition, weak gluteal muscles can also lead to impaired control of stability in the coronal and horizontal planes of the pelvis, triggering an abnormal patellar trajectory, increased patellofemoral joint stress, increased cartilage wear and an increased risk of PFPS injury.^[4,5]^

Neuromuscular electrical stimulation (NMES) is a method of using low-frequency pulsed electric current to directly stimulate nerves or muscles to enhance muscle function or treat neuromuscular disorders and injuries by passively inducing muscle contraction. It has been widely used in clinical neurology, neurorehabilitation and other fields as a painless and noninvasive treatment technology^.[6]^ Several physiological benefits of NMES have been reported, such as capillary dilation, and metabolic and neural recruitment changes. These changes are reflected in individuals in the form of decreased pain, increase functional and muscle recruitment edema.^[7]^ Some studies have reported the immediate effects of 4 weeks of a single patterned NMES treatment on pain and muscle activation in patients with PFPS, stimulating the VMO, GMD, hamstrings and adductor muscle groups, respectively, and found that only GMD activation was increased in patients with PFPS during the completion of 2 functional activities (one-legged shallow squat, side lean), with no difference in muscle activation in either the VMO or vastus lateralis (VL).^[8,9]^ There was an immediate decrease in pain levels, suggesting that the improvement in pain may be related to improved hip muscle activation, but not to improved peripatellar muscle activation. Previous studies have mainly utilized passive muscle stimulation as a single intervention to maintain or enhance muscle strength, but this approach has shown poor tolerance, difficulty, and limited efficacy. With the advancement of NMES technology, NMES devices can now provide synchronized muscle stimulation during movement therapy. Research has reported that combining NMES with functional training can effectively improve pain and lower extremity function.^[10]^ However, there is a lack of high-quality randomized controlled studies on the efficacy of NMES in PFPS rehabilitation, and contradictory results in the limited number of studies that warrant further exploration. To our knowledge, no studies have examined the effect of NMES combined with functional training on muscle recruitment.

### 1.1. Hypothesis section

The aim of our randomized controlled trial was to observe the effect of NMES combined with functional training on pain, lower extremity function and muscle recruitment in patients with PFPS.

## 2. Methods

### 2.1. Trial design

This study was a single-blind, randomized clinical trial. After screening, participants were randomized into 2 groups (control group and experimental group) through central allocation and consecutively treated for 6-weeks. Block randomization was performed using a computer-generated random number list prepared by a researcher with no clinical involvement in the trial. To maintain the objectivity of this study, we have separated the outcome assessor, clinical research coordinator, and therapist who treats the participant. In this study, individuals with conflicts of interest or preconceived positions are excluded to prevent related bias. This randomization process will be conducted by a third-party team of statisticians and the allocations will be concealed and sequentially numbered with opaque and sealed envelopes. Only the principle investigator will generate the allocation sequence, enrollment, and assign participants to interventions. The serial number codes will be opened in the presence of the participants and treatment team without assessor. And only the principle investigator will generate the allocation sequence, enrollment, and assign participants to interventions. Before conducting the study, ethical approval was obtained from the institutional ethics committee (assigned number 20200013), This study was in accordance with “The Code of Ethics of the World Medical Association (Declaration of Helsinki).” All participants were informed in detail about the study procedure, potential risks, and benefits.

### 2.2. Subjects

**Figure 1.**
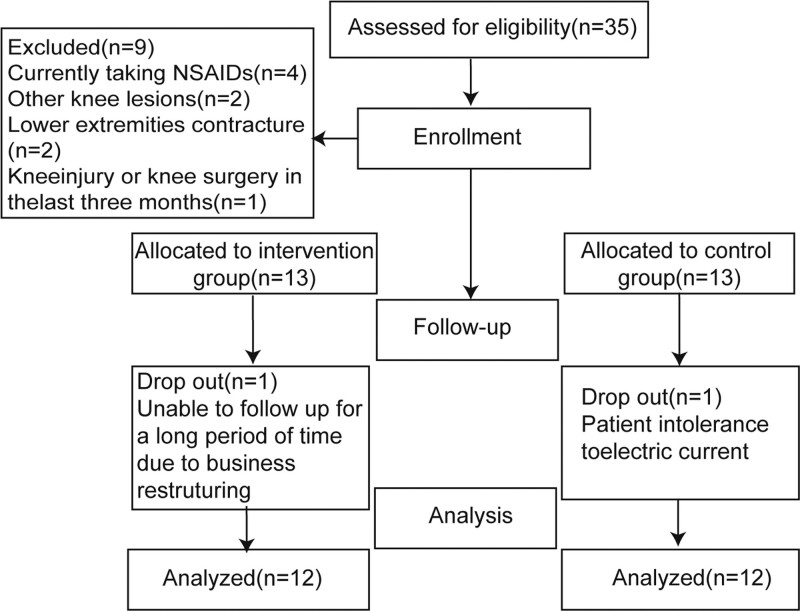
Consolidated Standards of Reporting Trials (CONSORT) chart showing recruitment and allocation of participants.

Sample size was determined based on previous studies, Motealleh et al^[11]^ in 16 patients with PFPS were treated with NMES for a period of 4 weeks, in which the visual analog scores (VAS), the main index of the test group, was (2.20 ± 2.15) before the intervention, and (0.71 ± 0.61) after the intervention, and the test level a was set to 0.05, and the test efficacy (1-β) was set to 0.80, and the minimum sample size of n = 18 was calculated, taking into account the 20% attrition rate, n ≈ 22 participants, with an allocation ratio of 1:1. A total of 35 patients diagnosed with PFPS were selected from the outpatient department of Sichuan Provincial Orthopedic Hospital between July 2020 and December 2020. Based on the inclusion and exclusion criteria, Excluded [n = 9, include Currently taking NSAIDs (n = 4), Other knee lesions (n = 2), Lower extremities contracture (n = 2), knee injury or knee surgery in the last 3 months (n = 1)]. Only 26 patients were included, and they were divided into the experimental and control groups. The allocation of participants into 2 groups is shown in Figure 1. The inclusion criteria were based on the PFPS clinical guidelines issued by the American Academy of Orthopedic Physical Therapy in 2019^.[8]^ The inclusion criteria were as follows: experiencing pain or exacerbation of pain during activities such as running, jumping, stair climbing, squatting, kneeling, etc; in 2 or more of the mentioned activities; positive results in the patellar tilt test, patellofemoral joint grinding test, resisted knee extension test, patellofemoral joint tenderness test, and palpation pain on the medial and lateral edges of the patella; age between 18 and 45 years; VAS score > 3 points; a history of anterior knee or peripatellar pain lasting for 3 days or more in the past 3 months (non-traumatic); no other lower limb knee joint injuries. The exclusion criteria were as follows: current use of non-steroidal anti-inflammatory drugs such as cortisone; other knee joint lesions or articular bone injuries; knee joint injury or surgery in the past 3 months; vestibular dysfunction; presence of internal metal fixation, etc.

### 2.3. Functional training

The control group underwent routine functional training, including warm-up activities, strength training, balance training, and relaxation exercises. The training progressed based on the patient condition, gradually increasing the duration or repetitions of the exercises. Each session lasted for 45 minutes, once a day, with training sessions scheduled every other day, 3 days/week for 6 weeks. The specific program was as follows: Warm-up activities, Muscle strength training, Balance training, Relaxation exercises (Table 1).

**Table 1:** Functional Training Plan.

Training content	Training type	Training frequency	Number of training groups	Interval time	Notes
Warm-up activities	Jogging Walking with a knee hugging motion	– 10 m away	– 1–3 group	3 min 15 s	
		Quadriceps stretching during walking	10 m away	1–3 group	15 s
Muscle strength training	Straight leg elevation with hip joint external rotation of 10°^[12]^	10 times	1–3 group	60 s	Advanced: Replace the elastic band with greater resistance, increase the duration or frequency of the action
	Clam opening and closing^[5,13]^	10 times	1–3 group	60 s	
	Squatting^[14]^	10 times	1–3 group	60 s	
	Double bridge movement^[15]^	10 times	1–3 group	60 s	
	Single bridge movement^[15]^	10 times	1–3 group	60 s	
Balance training	Standing with one leg open on flat ground Standing with one leg open on the BOSU ball^[5,12,16]^	2 min 2 min	1–3 group 1–3 group	30 s 30 s	Increase the number or duration of standing
	Standing with one leg and eyes closed on a BOSU ball^[5,13,16]^	2 min	1–3 group	30 s	
Relaxation exercises	Static stretching of Quadriceps, triceps surae, iliopsoas, hamstrings, gluteus, and relaxation of iliotibial band^[15]^	30 s	2 group	30 s	

### 2.4. Neuromuscular electrical stimulation

The quadriceps plays an important role in lateral patellar stabilization, and the VMO in particular is important in improving the VMO/VL balance and correcting the biomechanics of the patella in the trochanteric groove to reduce patellofemoral joint stress. The main role of the GMAX is in limiting hip adduction and internal rotation, maintaining normal lower extremity biomechanics, and thus correcting abnormal patellofemoral trajectories. Therefore, VMO and GMAX targeted stimulation were chosen in this study to improve PFPS. The Genesy 600 rehabilitation muscle electrical stimulation device (manufactured by GLOBU, Italy) was used for NMES treatment on the vastus medialis oblique (VMO) and GMAX muscles. During straight leg raising with hip external rotation and squatting training, the NMES electrodes were attached to the VMO to activate the VMO. The VMO electrode was placed 4 cm above and 3 cm medial to the patella, at a 55° angle relative to the femur longitudinal axis.^[17]^ During clam opening and closing, single and double bridge training, the NMES electrodes were attached to the GMAX to activate GMAX, the GMAX electrode was placed at the midpoint of the muscle belly in the line between the second sacral vertebra and the greater trochanter of the femur,^[18,19]^ and the muscles contracted during training when an electric current passed through them, and rested when the electric current ceased. In order to avoid the muscle fatigue. Two electrode pads were placed on the marked areas of VMO and GMAX using the “side-by-side” method, with a center distance of 2 cm between the electrode pads and aligned with the direction of muscle fibers. The selected stimulation mode was “muscle electrical stimulation” with a frequency of 20 Hz, pulse width of 200 μs, intensity of 10 to 20 mA, duty cycle of 1:2, 20 minutes per session, training every other day, 3 days per week. The output current intensity was adjusted to elicit maximal muscle contraction without causing significant discomfort.^[20]^ Due to the noticeable increase in threshold after 2 weeks of intervention, the stimulation intensity needed to be appropriately increased.

### 2.5. Outcome measurements

To assess the impact of the intervention on pain and function, a VAS with a length of 10 cm was used to evaluate the severity of knee joint pain in patients. The scale ranged from 0 to 10, with 0 indicating no pain and 10 indicating unbearable pain.

The Kujala Rating Scale is internationally recognized as the most commonly used tool for evaluating knee pain and function.^[21]^ It can effectively assess patient function with an ICC of 0.90 to 0.98 The Kujala Scale is recognized internationally as the most commonly used instrument for assessing knee pain and function. It consists of 13 items, limping, weight bearing, walking, the maximum score for each item was 5 points for limping, weight bearing, walking, thigh muscle atrophy, limited flexion and squatting; 5 points for walking up and down stairs, running, jumping, prolonged sitting in a bent knee position, pain, and the ability to walk.^[22]^ The maximum score for each item was 10 points for walking up and down stairs, running, jumping, prolonged sitting in a bent-knee position, pain, swelling, and patellar subluxation. The Kujala score was employed to evaluate knee joint function, with a maximum score of 100. A higher score indicates better knee joint function, whereas a lower score reflects more severe impairment.

To test muscle signals, Mega 6000 type 16-channel surface electromyography (EMG) device was used to measure the surface EMG signals of VMO, VL, and VMO/VL during squat exercises. Electrode placement was guided by a reference line connecting the anterior superior iliac spine and the center of the patella. The target muscles were identified by manual resistance and marked for electrode placement. VMO electrode placement was the same as NMES. The VL electrode was placed 10 cm above and 6 to 8 cm lateral to the patella, at a 15° angle relative to the femur longitudinal axis.^[17]^ The reference electrode was placed approximately 2 to 3 cm adjacent to the testing electrodes to prevent displacement. Prior to electrode placement, the area of the target muscles was shaved, exfoliated, and sanded smooth with fine sandpaper. Then, the area was disinfected with 75% alcohol, ensuring local dryness before applying the electrode pads. The Mega Win testing software was opened, and subject information and test protocols were created. The surface EMG signals were recorded when the subject was in a static state, observing a stable baseline signal for 3 to 5 seconds. The subject was instructed to perform squatting movements, and the EMG signals were continuously recorded. The subject stood with hands on hips and feet shoulder-width apart. Upon receiving the command, the subject performed squatting movements at a moderate speed (guided by a metronome set at 2-second intervals), flexing the hip joint before flexing the knee joint. The instructions given by the tester were as follows: “Ready” to prepare the subject, “1” to start hip flexion to the maximum extent, and “2” to start squatting to a 60° angle, maintaining it for 5 seconds. This was repeated twice. Then, the subject stood up at a moderate speed (2 seconds) and repeated the entire sequence twice. The Mega Win 3.01 signal processing software was used for analysis and processing. All EMG signals were amplified, band-pass filtered (20–500 Hz), smoothed, and sampled at 1000 Hz. Data segments with higher amplitudes and minimal interference lasting for 2 seconds were selected to calculate average root mean square (RMS) and average integrated electromyography (IEMG) for further analysis. In this study, RMS and IMEG were selected as indicators for efficacy evaluation. RMS amplitude reflects the average effect of EMG signals over a period of time, i.e., muscle contraction strength. It is generally considered to be related to the synchronization of motor unit recruitment and excitatory rhythms, and can be used as a test index for assessing the state of muscle atrophy and the size of muscle strength, and is the most commonly used index in the analysis of surface EMG time-threshold indexes.^[23]^The IEMG refers to the total sum of motor unit discharges generated by muscle activity in a specific period of time, and the size of the IEMG reflects the number of motor units recruited at the same time as well as the participation in each motor unit discharge size.^[24]^ Therefore, RMS and IEMG can be used as an indirect indicator of muscle strength size. In this study, the EMG data of the EMG curves of the muscles measured in the lower limbs during the squatting movement were first subjected to full-wave rectification and then to superimposed averaging, and the superimposed averaging process was mainly the average value taken, so the calculation formula: average IEMG value = (IEMG 1st + IEMG 2nd)/2.

### 2.6. Statistical analyses

IBM Statistical Package for Social Sciences (IBM SPSS) version 20 for Windows was used for statistical analysis. The data were checked for missing data, outliers, and normal distribution. Normal distribution was assessed using the Shapiro–Wilk test of normality for all outcome variables. Data were identified as not normally distributed. As a result, nonparametric tests were performed for further data analysis. The within-group comparison was determined using the Wilcoxon signed-rank test for differences in Kujala scores and VAS scores before and after intervention, while the between-group comparison was determined using the Mann–Whitney *U* test for comparison of differences in height, disease duration, Kujala score and VAS score between the 2 groups. The chi-square test was used to compare the gender of the 2 groups. A 2 × 2 mixed design ANOVA was used to determine the effect of time (pre-intervention vs post-intervention) and grouping (experimental group VS control group) on VAS, Kujala, and EMG in patients with PFPS. If there was an interaction between time and group, Bonferroni post hoc test was applied, and the effect size was calculated using Cohen d s for post hoc comparisons, with d = 0.2 considered as small-, d = 0.5 as a medium-, and d = 0.8 as a large effect size. The significance level was established at 0.05, with a 95% confidence interval.

## 3. Results

### 3.1. Subjects baseline data

During study period, no AEs related to our study was observed in either group. There was one case of lost visit in each of the experimental and control groups, and the lost visits are shown in Figure 1. Only 24 patients were finally included in the outcome analysis. Subjects’ baseline demographics and clinical characteristics are shown by mean standard deviation in Table 2. Both NMES and control groups were composed of 24 participants. The 2 groups were well matched at baseline assessment. There was no statistically significant intergroup differences for any parameter analyzed. Indicating comparability (Table 2).

**Table 2:** Comparison of general information of patients in 2 groups.

Group	Sex (m/f)		Age (yr)		Weight (kg)	Height (cm)	Duration (mo)
	Male	Female	Mean ± SD		Mean ± SD	Mean ± SD	Mean ± SD
Experimental group	7	5	32.83 ± 8.55	65.96 ± 10.99		168.58 ± 5.40	8.87 ± 9.24
Control group	6	6	36.33 ± 6.58	64.50 ± 7.30		167.33 ± 6.79	9.67 ± 7.87
*t*/*U*/*χ*^2^	0.17		−0.38	1.12		−1.36	−1.08
* P* value	.68		.71	.27		.18	.29

Values are presented as mean (standard deviation). Control group: functional training, experimental group: neuromuscular electrical stimulation.

### 3.2. VAS and Kujala scores

Table 3 shows there were significant reductions in VAS scores [control group (z = −2.98, *P* = .00), experimental group (z = −2.97, *P* = .00)], and VAS scores was reduced more in the experimental group compared to the control group (U = −2.25, *P* = .00).

**Table 3:** Comparison of VAS pre- and post-intervention.

group	Number	VAS (scores) pre-intervention	VAS (scores) post-intervention	Z value	*P* value
Experimental group	12	4.58 ± 1.51	0.75 ± 0.97	−2.97	<.001**
Control group	12	4.42 ± 0.99	1.42 ± 0.51	−2.98	<.001**
U value		−4.62	−2.25		
* P* value		.64	.02*		

* Statistically significant (*P* < .05) difference between control group and experimental group.

** (*P* < .001) Statistically highly significant difference between before and after intervention.

VAS = visual analog scores.

Table 4 shows there were significant reductions in Kujala scores [control group (z=−2.81, *P* = .00), experimental group (z = −2.94, *P* = .00)], and Kujala scores was increased more in the experimental group compared to the control group (U = −2.28, *P* = .00).

**Table 4:** Comparison of Kujala pre- and post-intervention.

Group	Kujala (scores) pre-intervention	Kujala (scores) post-intervention	Z value	*P* value
Experimental group	73.25 ± 6.64	90.08 ± 5.73	−2.94	<.001**
Control group	74.83 ± 11.77	85.17 ± 2.12	−2.81	<.001**
U value	−1.20	−2.28		
* P* value	.23	.02*		

* Statistically significant (*P* < .05) difference between control group and experimental group.

** (*P* < .001) Statistically highly significant difference between before and after intervention.

### 3.3. EMG results

There was an interaction effect of time with grouping on RMS of VMO (*P* = .02), which showed that both experimental and control groups had an increase in muscle contraction capacity in the intervention compared to pre-intervention (*P* = .00) Cohen d experimental group = 2.96, Cohen d control group = 1.05, whereas there was no statistical difference between the 2 groups (*P* = .34). There was an interaction effect of time and group on IEMG of VMO (*P* = .02), which showed that both experimental and control groups increased muscle recruitment compared to pre-intervention (*P* = .00, Cohen d experimental group = 2.56, Cohen d control group = 1.38) while there was no statistically significant difference between the 2 groups (*P* = .05) (Table 5).

**Table 5:** Comparison of RMS and IEMG pre- and post-intervention.

	Group	Pre-intervention (mean ± SD)		Post-intervention (mean ± SD)	
		RMS (μV)	IEMG (μVs)	RMS (μV)	IEMG (μVs)
VMO	Experimental group	60.58 ± 7.11	4.92 ± 1.08	77.00 ± 3.30**	7.08 ± 0.51**
	Control group	61.25 ± 10.51	4.83 ± 1.03	70.92 ± 7.73**	6.00 ± 0.60**
VL	Experimental group	75.92 ± 10.16	6.25 ± 1.29	75.08 ± 8.79	7.00 ± 0.95
	Control group	75.08 ± 11.02	6.33 ± 1.23	76.00 ± 7.73	6.87 ± 0.61

** (*P* < .001) Statistically highly significant difference between before and after intervention.

IEMG = integrated electromyography, RMS = root mean square, VL = vastus lateralis, VMO = vastus medial oblique.

There was no interaction between time and grouping on RMS of VL (*P* = .63) and the results showed no change in muscle contraction capacity of VL in the experimental and control groups (*P* = .99). There was no interaction between time and grouping on IEMG of VL (*P* = .72) and the results showed no change in muscle recruitment capacity of VL in the experimental and control groups (*P* = .95) (Table 5).

There was an interaction between time and grouping on the RMS of the VMO/VL ratio (*P* = .00), which showed that both experimental and control groups improved the balance of VMO/VL contractility more after the intervention than before (*P* = .00, Cohen d experimental group = 2.60, Cohen d control group = 1.38), and that the experimental group had a higher RMS of VMO/VL than the control group (*P* = .01, Cohen d post-intervention = 1.47). Time in grouping interacted with both VMO/VL IEMG (*P* = .04), with both experimental and control groups improving the balance of VMO/VL muscle recruitment capacity more than pre-intervention (*P* = .00, Cohen d experimental group = 1.86, Cohen d control group = 1.05), and the experimental group VMO/VL IEMG being higher than the control group (*P* = .01, Cohen d post-intervention = 1.27) (Tables 6).

**Table 6:** Comparison of VMO/VL ratio pre- and post-intervention.

group	Pre-intervention (mean ± SD)		Post-intervention (mean ± SD)	
	RMS (%)	IEMG (%)	RMS (%)	IEMG (%)
Experimental group	0.80 ± 0.07	0.78 ± 0.09	1.05 ± 0.11**^,^*	1.03 ± 0.15**^,^*
Control group	0.81 ± 0.07	0.76 ± 0.06	0.92 ± 0.06**	0.87 ± 0.09**

* Statistically significant (*P* < .05) difference between control group and experimental group.

** (*P* < .001) Statistically highly significant difference between before and after intervention.

IEMG = integrated electromyography, RMS = root mean square, VL = vastus lateralis, VMO = vastus medial oblique.

## 4. Discussion

In this study, we found that NMES combined with functional training and functional training alone were effective in improving muscle recruitment, decreasing pain symptoms, and improving the level of knee function in patients with PFPS, and that NMES combined with functional training was more effective in improving knee function.

Although the pathogenesis of PFPS remains unclear, patients exhibit greater patellofemoral joint stress compared to individuals without pain.^[25]^ Excessive patellofemoral joint stress may disrupt joint homeostasis, which may irritate periarticular neural structures such as cartilage, medial and lateral supportive bands, and the infrapatellar fat pad, which in turn induces pain. Joint pain affects physical activity, and reduced physical activity in turn leads to reflex inhibition, VMO muscle atrophy, and reduced muscle strength, creating a vicious cycle that further exacerbates pain and continues to degrade knee function, causing long-term discomfort and severely affecting patients’ daily activities and quality of life.

In this study, we found that 6 weeks of NMES combined with functional training had a certain effect on reducing pain symptoms and improving knee function in patients with PFPS compared with functional training alone, and that NMES combined with functional training was more effective in improving the level of knee function. This is consistent with the study by Das^[26]^ and Bily.^[27]^ Bily found that both a single supervised exercise training and electrical stimulation (using 40 Hz, pulse duration of 0.26 ms, duty cycle of 5:10, duration of 20 minutes, 2 times per week for a total of 12 weeks) combined with exercise training can significantly reduce pain and improve function,^[27]^ Das also Compared with a single supervised exercise program, a 4-week combined NMES (using a frequency of 30 to 75 Hz, pulse width of 20 to 1000μs, duty cycle of 10:50, and a duration of 30 minutes with biphasic rectangular pulses) and supervised exercise training program can effectively alleviate pain and improve functional impairment in PFPS patients. It is believed that the mechanism of pain relief by NMES may be related to its ability to selectively stimulate the VMO, increase neural recruitment to the VMO muscle, increase stimulation of A-α and A-β fibers that do not transmit nociception, inhibit nociceptive A-δ and C fiber conduction, improve the VMO/VL balance, correct patellar trajectory malpractice, and break the vicious circle of pain-muscle atrophy-pain to alleviate pain. The possible mechanism of functional training for pain relief is related to the rhythmic muscle contraction during training, which regulates the release of B-lipotropic hormone from the pituitary gland by stimulating A-δ fibers, which is further broken down into β-endorphins, resulting in the reduction of pain symptoms.^[28]^ In conclusion, NMES combined with functional training may have a more positive effect than functional training alone in reducing pain and improving knee function.

The results of this study showed that the RMS and IEMG of VMO and the RMS and IEMG of VMO/VL ratio were significantly elevated in both groups of subjects after the intervention, which suggests that the functional training can effectively improve the VMO muscle recruitment and enhance the VMO and VL balance; the RMS and IEMG of VMO was no statistically difference between the 2 groups, but the experimental group was significantly higher than the control group and the RMS and IEMG of VMO/VL ratio in the subjects in the experimental group were significantly higher than that in the control group after the intervention, which suggests that NMES treatment helps to further improve VMO muscle recruitment to increase the relative neuromuscular electrical activity ratio of VMO/VL and restore VMO/VL balance.

A large number of previous studies have confirmed that the phenomenon of “knee weakness” in PFPS patients is related to quadriceps atrophy. In particular, atrophy of the VMO or relative weakness of the VL can lead to abnormal patellar trajectory, reduce the contact area of the patellofemoral joint, increase the pressure on the patellofemoral joint, and cause patellofemoral joint dysfunction.^[29,30]^ In healthy subjects, the ideal ratio of EMG for VMO/VL balance is 1.^[31]^ Some studies have found that VL activation was higher than VMO in PFPS patients compared to healthy subjects, and there was a severe muscle imbalance between VMO and VL. Studies have shown that functional training is beneficial in enhancing VMO muscle recruitment and improving VMO/VL balance in PFPS patients. The results of this study showed that after 6 weeks of intervention treatment, the RMS and IEMG values of VMO and VMO/VL ratio were significantly higher in both groups, with better improvement in the experimental group compared with the control group, suggesting that NMES helps to further promote the recruitment of VMO muscles in PFPS patients.

The results of this study showed that after 6 weeks of intervention treatment, the RMS and IEMG values of VMO and VMO/VL ratio were significantly higher in both groups, with better improvement in the experimental group compared to the control group, suggesting that NMES helps to further promote the recruitment of VMO muscles in patients with PFPS. MES optimizes the recruitment of its motor units by providing targeted stimulation of the VMO, improving selective inhibition of motor units and the autonomic control of muscle activation during functional movements. This may involve an increase in corticospinal excitability due to altered plasticity of the type Ia reflex pathway, which in turn allows for a more comprehensive recruitment of motor units, improves VMO muscle contraction, improves the relative neuromuscular ratio of the VMO/VL in patients with PFPS, restores the VMO/VL balance, and corrects aberrant neuromuscular control and movement patterns, and may be the reason why electrical stimulation in combination with functional training is so effective at improving muscle recruitment and alleviating pain. This may also be one of the mechanisms by which electrical stimulation combined with functional training can effectively reduce improve muscle recruitment and relieve pain.^[7]^

In addition, there was a lack of improvement in VL IEMG findings in this study. Garcia et al^[32]^ monstrated that electrical stimulation of the VMO using bi-directional low-frequency pulses with pulse duration of 250 to 500 µs, frequency of 30 to 50 Hz, and on/off ratios of 6 to 10:10 to 50, 3 times a week for 12 to 15 contractions, for a period of 4 to 6 weeks, and found that NMES treatment could effectively activate the VMO, inhibit the VL, and improve the VMO/VL balance (IEMG of VMO/VL increased from 0.89 ± 0.30 to 1.82 ± 0.69). This is consistent with the results of the present study. This may be due to the fact that NMES stimulated the VMO directly and did not act directly on the VL, which may have led to an increase in the degree of nerve recruitment to the VMO and a lack of muscle recruitment to the VL, which in part explains the improvement in VMO muscle recruitment in the present study and the lack of significant differences in the VL IEMG signals.

Currently, most studies have focused on single electrical stimulation of VMO, while there is limited research on using NMES for stimulating the GMAX. However, multiple studies have shown a close correlation between the occurrence of PFPS and gluteal muscle dysfunction. The latest clinical guidelines state that gluteal muscle weakness may be a result of PFPS, suggesting that gluteal muscle inhibition may be a result of long-term poor function and altered movement patterns.^[33]^ Analysis of its mechanism suggests that the weakness of the GMAX, which plays a role in hip abduction and external rotation, can lead to impaired control of pelvic coronal and horizontal stability. This can result in abnormal patellar movement trajectory, increased patellofemoral joint pressure, aggravated cartilage wear, and increased risk of PFPS injury.^[29]^ Therefore, the use of NMES to activate the GMAX holds practical significance for improving clinical symptoms of PFPS.^[34,35]^

In summary, NMES combined with functional training can further enhance the recruitment of VMO muscle, improve VMO/VL balance, and be more beneficial in reducing pain and improving lower extremity function and muscle recruitment in PFPS patients. NMES is considered an adjunctive therapy supplemented with different interventions to improve muscle strength and function. The present study was conducted to produce an additional impact of NMES treatment superimposed on functional training, which may help in future studies using different NMES synchronized with other interventions to enhance the rehabilitation efficacy of PFPS patients through passive versus active stimulation.

In this study, no severe adverse reactions were observed in any of the patients. NMES is simple to operate, safe, and allows targeted muscle stimulation during movement to maximize muscle activation, making it worthy of clinical promotion. The limitations of this study include the lack of a single electrical stimulation treatment as a control group, focusing only on observing the efficacy of 6 weeks of NMES combined with functional training in PFPS patients, and the failure to track its long-term effects. Future high-quality randomized controlled trials should be conducted to further clarify its effectiveness.

## 5. Limitations

Due to the limitation of study time and study conditions, a single electrical stimulation treatment was not set as a control group, and the results of the study may be biased. The study only focused on the efficacy of 6 weeks of NMES combined with functional training in patients with PFPS, but failed to track its long-term efficacy. Due to the financial and time constraints of this study, the sample size was small and subgroup analyses of age were not performed. In the future, further comparisons of the efficacy of patients of different ages can be carried out, which is a shortcoming of this study.

## 6. Conclusion

Both functional training and NMES are effective in enhancing muscle recruitment and improving pain and knee function in patients with PFPS, and the combination of the functional training and NMES is more effective.

## Acknowledgments

The authors thank Sports medicine Department Outpatient Department of Sichuan Orthopaedic Hospital for their help in preparing the article. The authors also thank the subjects for their time and effort.

## Author contributions

**Conceptualization:** Qiu Nie, Yaming Yu, Zheng Yuan, Jian Chen.

**Data curation:** Qiu Nie.

**Formal analysis:** Qiu Nie, Jian Chen.

**Investigation:** Qiu Nie, Yaming Yu, Zheng Yuan, Jian Chen.

**Methodology:** Qiu Nie.

**Project administration:** Qiu Nie, Jian Chen.

**Resources:** Qiu Nie.

**Software:** Qiu Nie.

**Supervision:** Qiu Nie, Yaming Yu, Zheng Yuan, Jian Chen.

**Validation:** Qiu Nie, Yaming Yu, Zheng Yuan, Jian Chen.

**Visualization:** Qiu Nie, Zheng Yuan, Jian Chen.

**Writing – original draft:** Qiu Nie, Yaming Yu, Zheng Yuan, Jian Chen.

**Writing – review & editing:** Qiu Nie, Yaming Yu, Zheng Yuan, Jian Chen.
